# Innovative Approaches to Improve COVID-19 Case Investigation and Contact Tracing Among Refugees, Immigrants, and Migrants: Lessons Learned from a Newly Established National Resource Center

**DOI:** 10.1007/s10903-023-01508-y

**Published:** 2023-06-07

**Authors:** Erin M. Mann, Michelle Weinberg, Elizabeth Dawson-Hahn, Sarah K. Clarke, Madison Olmsted, Nathan Bertelsen, Ridhi Arun, Megan Keaveney, Shanna Miko, Amy Kircher, Anna E. Pendleton, Brett Hendel-Paterson, Shailendra Prasad, William M. Stauffer

**Affiliations:** 1 National Resource Center for Refugees, Immigrants, and Migrants, University of Minnesota, 420 Delaware St SE, Minneapolis, MN 55455 USA; 2 Center for Global Health and Social Responsibility, University of Minnesota, Minneapolis, MN USA; 3 Immigrant, Refugee and Migrant Health Branch, Division of Global Migration and Quarantine, Centers for Disease Control and Prevention, Atlanta, GA USA; 4 Department of Pediatrics, University of Washington, Seattle, WA USA; 5 International Rescue Committee, Dallas, TX USA; 6 Center for State, Tribal, Local, and Territorial Support, Centers for Disease Control and Prevention, St. Paul, MN USA; 7 University of Minnesota Medical School, Minneapolis, MN USA; 8 IDEO.org, San Francisco, CA USA; 9 Strategic Partnerships and Research Collaborative, University of Minnesota, Minneapolis, MN USA; 10 Department of Medicine, University of Minnesota, Minneapolis, MN USA

**Keywords:** Contact tracing, Refugees, Immigrants, Migrants, COVID-19

## Abstract

Effective COVID-19 case investigation and contact tracing (CICT) among refugee, immigrant, and migrant (RIM) communities requires innovative approaches to address linguistic, cultural and community specific preferences. The National Resource Center for Refugees, Immigrants, and Migrants (NRC-RIM) is a CDC-funded initiative to support state and local health departments with COVID-19 response among RIM communities, including CICT. This note from the field will describe NRC-RIM and initial outcomes and lessons learned, including the use of human-centered design to develop health messaging around COVID-19 CICT; training developed for case investigators, contact tracers, and other public health professionals working with RIM community members; and promising practices and other resources related to COVID-19 CICT among RIM communities that have been implemented by health departments, health systems, or community-based organizations.

## Introduction

More than 45 million people living in the United States were born in another country [1]. These individuals represent many cultures, languages, and motivations for migration. Multiple factors increase the risk of acquiring COVID-19 and developing severe COVID-19 among refugees, immigrants, and migrants (RIM) [2]. Factors that place RIM communities at higher risk of acquiring COVID-19 also impact the effectiveness of public health interventions such as case investigation and contact tracing (CICT). For example, multigenerational households may pose challenges for quarantine and isolation; some community members may fear immigration enforcement and distrust government agencies, inhibiting the collection of case and contact information; and some community members may have limited English proficiency, requiring an interpreter or bilingual case investigator or contact tracer to ensure effective interviews [3].

The National Resource Center for Refugees, Immigrants, and Migrants (NRC-RIM) was established to develop and disseminate practical resources for health departments and community organizations to use in their work with RIM communities disproportionately affected by COVID-19. Recognizing that some jurisdictions and organizations have had success serving and partnering with RIM communities, NRC-RIM is a venue through which timely and practical information and resources can be shared with those facing challenges. In this note from the field, we describe NRC-RIM activities related to CICT among RIM communities and share outcomes and lessons learned. While NRC-RIM refers to RIM communities collectively, we recognize the significant differences that exist across many RIM communities. Our resources aimed to address common gaps affecting these communities and whenever possible, our work was done in partnership with community members and resources were developed that would allow individual organizations to customize and adapt based on their local needs.

## Background

NRC-RIM was established in October 2020 at the University of Minnesota with funding from the US Centers for Disease Control and Prevention and the International Organization for Migration [4]. Partners include IDEO.org, International Rescue Committee, Migrant Clinicians Network, Minnesota Department of Health, and National Association of County and City Health Officials. While these partners were engaged to support the development of these materials, the audience of NRC-RIM is much broader and all resources are freely available to any jurisdiction, community-based organization, health systems provider, or other organization that finds the resources useful. NRC-RIM resources and activities include promising practices, health communications and health education, and training for public health professionals. Promising practices are strategies, approaches, or programs that have anecdotally shown to have a positive impact in some local settings.

An early focus for NRC-RIM was CICT, given known challenges conducting CICT among RIM communities. NRC-RIM and partners reviewed available resources and performed listening sessions and interviews with public health professionals, community members, and healthcare providers. Several key themes emerged, including the importance of community engagement and partnerships to encourage participation and trust in CICT, hiring bilingual and bicultural staff from local communities to serve as CICT professionals and/or using professional interpreters, approaching CICT encounters with an understanding that some RIM communities may have well-founded mistrust of government authorities, supporting CICT professionals with training related to working with RIM communities, developing culturally and linguistically appropriate messaging about CICT, and partnering with local community organizations to provide social services needed during isolation and quarantine. Opportunities were identified to develop new resources, particularly health communications and health education, training, and a toolkit specific to conducting CICT among RIM communities. NRC-RIM activities were determined to be non-human subjects research by the University of Minnesota institutional review board.

## Results

### CICT Health Communications and Health Education

NRC-RIM reviewed available resources about CICT and identified a dearth of culturally and linguistically appropriate materials, particularly video and audio resources. Existing resources did not feature RIM community members and most resources appeared to have limited community input. NRC-RIM engaged in several CICT health communications and health education efforts to address these gaps.

#### Translated Materials Library

NRC-RIM created a library of publicly available multilingual COVID-19 health communications and health education materials, leveraging collection efforts of the Washington State Department of Social and Health Services Office of Refugee and Immigrant Assistance [5]. NRC-RIM compiled resources from health departments, community-based organizations, and health clinics and organized materials by language, resource type, and topic. A total of 6000 individual resources were added to the library through June 2021. Following a comprehensive search, fewer than 200 of the total resources in the library were about CICT. Often there was only one CICT resource per language among languages commonly spoken by RIM communities in the US.

#### Community Videos: “What is a Contact Tracer”

In partnership with the International Rescue Committee (IRC), NRC-RIM developed a series of videos explaining the role of contact tracers. IRC hired a team of bilingual, bicultural content validators from several refugee and immigrant communities. Videos were produced in multiple languages and featured a community member describing the work of a contact tracer to prepare community members if they receive a call from the local public health department. Communities and languages were selected to reflect the largest refugee communities in the United States. IRC worked with community members to develop the script and ensure the language was simple, clear, and addressed potential concerns around receiving a contact tracing call. For example, the video focuses on a contact *tracer* (the individual professional making the call) rather than contact *tracing* (the act of contact tracing) based on feedback from community members that this approach was less threatening. The videos were released in early 2021 on the NRC-RIM website and NRC-RIM YouTube channel. The videos were also distributed through IRC’s network of local offices via WhatsApp, text message, and email to community leaders.

#### Community-Led CICT Messaging

**Fig. 1:**
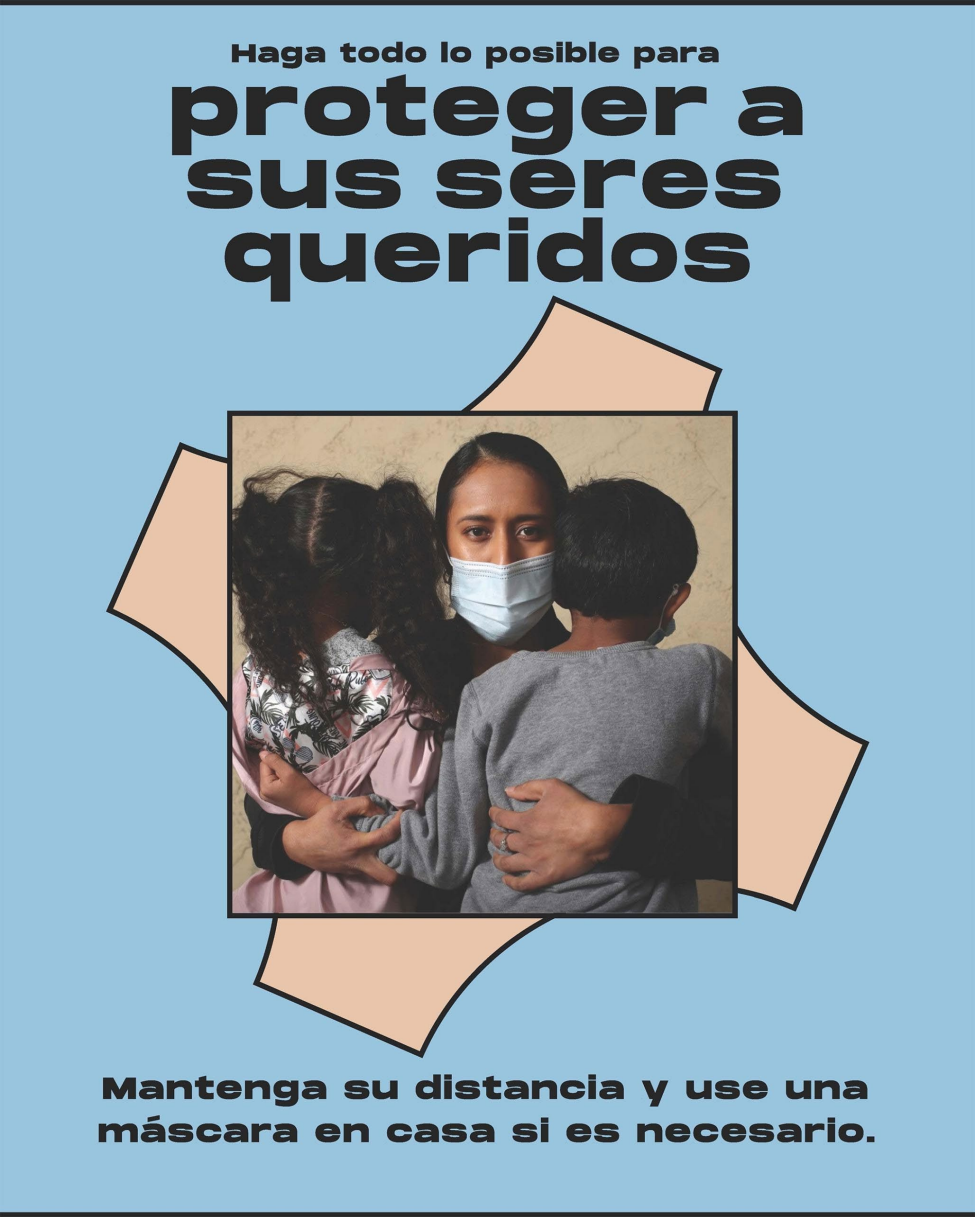
Visual example of CICT Campaign focused on safety and increasing protection. Note: “English translation (top to bottom): Do all you can to protect your loved ones. Keep your distance and wear a mask at home if you have to.”

It is well-understood that health messaging should be linguistically and culturally appropriate and developed in partnership with communities, however creation of materials often centers the perspective of public health professionals rather than the communities. NRC-RIM partnered with IDEO.org, a non-profit design studio specializing in human-centered design (HCD) to co-create campaigns with RIM communities to build awareness and willingness to participate in CICT. HCD is a problem-solving approach that starts with gaining empathy for one’s audience and concludes with solutions tailored to meet their needs. Messaging was created in partnership with a community of farmworkers in Florida, a community of Congolese refugees in Texas and a Hmong community in Minnesota. These communities were engaged to reflect a wide diversity of perspectives. Alongside community leaders and members, IDEO.org facilitated activities to understand concerns, misconceptions, and opportunities regarding COVID-19 and CICT in their respective communities, and co-designed materials that could address specific fears and misconceptions that were culturally relevant and unique to each community. By engaging community leaders, the campaigns could speak authentically to specific concerns held in each community; the language, tone, and visual style was unique and customized; and community members could feel ownership of the materials created. [Insert Table 1; Fig. 1.] A guidebook and visual kit were also developed, allowing communities to develop their own CICT campaigns.

**Table 1:** Summary of contact tracing campaigns co-created with refugee, immigrant, and migrant communities

Campaign	Purpose	Example Messages
Safety and increasing protection	Ensure community members have workarounds to reduce the spread of COVID-19 in the absence of resources or infrastructure for contact tracing	“Our collective wellbeing is in our hands.” “Do all you can to protect your loved ones.”
Awareness of COVID-19 contact tracing	Build an understanding of what contact tracing is and is not, and what role it has in preventing the spread of COVID-19	“Contact tracing can protect our community.” “[Contact tracing] allows us to know who else might need to get tested so we can stop the spread.”
Willingness to participate in contact tracing	Instill trust and confidence that drive individuals to action and opting in to participate in contact tracing.	“To disclose symptoms is an act of love.” “To notify others is an act of love.” “Save this number. If you’re sick, disclose your symptoms. If you test positive, self-isolate. If you’ve been in close contact with others, allow your health department to anonymously notify them.”

**Fig. 2:**
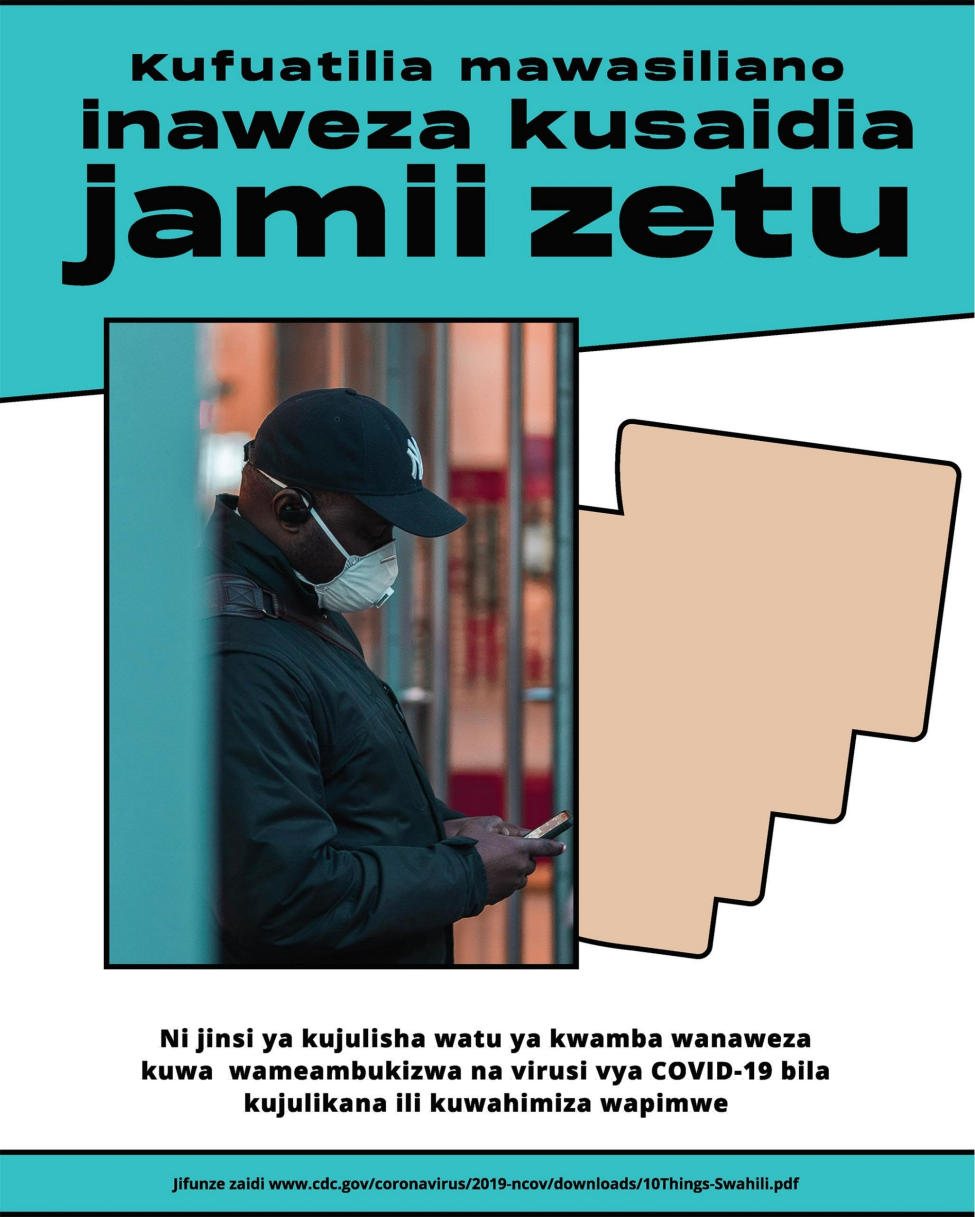
Visual example of CICT campaign focused on awareness of COVID-19 contact tracing. Note: English translation (top to bottom): contact tracing can help our community. It’s an anonymous way to tell others that they might have been exposed to COVID-19 and encourage them to test. Learn more [URL]

**Fig. 3:**
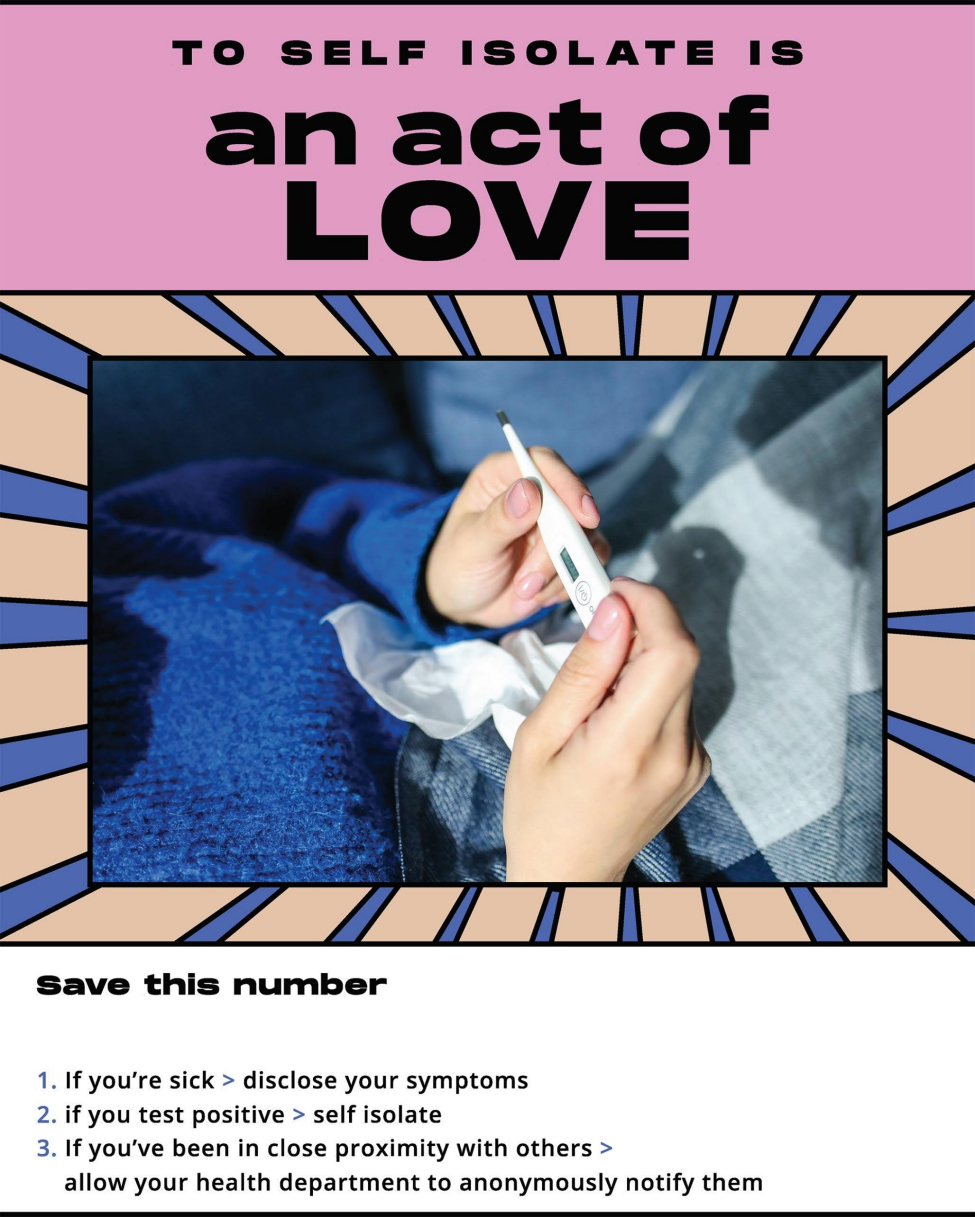
Visual example of CICT campaign focused on willingness to participate in COVID-19 contact tracing.

### Training for Public Health Professionals

After reviewing publicly available training resources, NRC-RIM held listening sessions with case investigators and contact tracers to inform curriculum development. NRC-RIM found a lack of material addressing skills and knowledge specific to working with RIM communities during COVID-19. Based on these findings, NRC-RIM developed new training modules to address language barriers, and to develop awareness and skills around cultural humility and social-determinants of health.

NRC-RIM worked with IRC to develop an interactive, scenario-based module on working with interpreters during CICT. The module covers topics related to techniques for working with interpreters virtually, solutions to challenges faced when working with cases/contacts and interpreters, and methods to improve the quality of an interpreted conversation. IRC created a supplemental glossary of CICT terms in English that was translated into multiple languages for interpreters working with case investigators and contact tracers.

Additionally, recognizing that case investigators and contact tracers were undergoing tremendous stress due to working in isolation, encountering cases/contacts during a personal or family health crisis, and the overwhelming demand for their services, NRC-RIM developed a self-care and resilience module and an accompanying training for supervisors. CICT professionals based in Minnesota were engaged to develop the training content, but the training resource was made available to all jurisdictions. The training includes real case investigators and contact tracers discussing their own stories, stressors, and coping mechanisms. An accompanying self-care guide addresses stress, secondary stress, burnout, resilience, and mindfulness, along with resources to cope with stress and promote self-care and well-being. The facilitator’s guide for supervisors provides practical recommendations for conducting listening sessions and suggestions for supporting staff.

### Practical Resources for Working with RIM Communities

Through interviews with public health professionals, community leaders, and healthcare providers, NRC-RIM identified many practical strategies, approaches, or programs to improve the effectiveness of CICT among RIM communities. To disseminate findings and amplify local successes, NRC-RIM developed a CICT toolkit intended for health departments across the country. The toolkit contains practical, concise, and instructive materials that health departments can implement. The toolkit contains a collection of practical tips for CICT among RIM communities (e.g. collecting preferred language at testing locations and pre-arranging an interpreter during a case investigation) and checklists of steps health departments can take to organize social services to help community members follow CICT-related public health guidance.

The toolkit also contains promising practices from health departments and community-based organizations - strategies, approaches, or programs that have anecdotally shown to have a positive impact in local settings. For example, the use of WhatsApp (a social messaging phone app) to engage RIM communities ahead of a CICT call and support groups for CICT teams in the form of multilingual “virtual coffee hours.” The toolkit also includes guides that illustrate practical steps of implementing new elements to CICT work such as a guide to translating and culturally adapting CICT interview questions about sexual orientation and gender identity.

#### Dissemination and Evaluation

All of the resources described above were made freely available on the NRC-RIM website and were promoted widely through NRC-RIM’s networks. Given that access to the NRC-RIM website is free and does not require users to register for access, detailed metrics regarding which organizations accessed the materials are not available. However, high level metrics regarding utilization are available. CICT-related content on the NRC-RIM website (toolkits, guides, health communications) received over 7,000 web page views with visitors from all 50 states and DC. Table 2 highlights several of the most popular CICT-related resources.

**Table 2:** Examples of contact tracing resources developed by NRC-RIM

Name of Resource	Description	URL
CICT Toolkit	This toolkit contains practical resources for public health departments to support CICT among refugee, immigrant, and migrant communities.	https://nrcrim.org/toolkits/case-investigation-contact-tracing
CICT Glossary of Terms	This glossary is for interpreters providing interpretation for COVID-19 mitigation and prevention efforts, such as those working with public health departments, contact tracers and case investigators. Available in English, Arabic, Burmese, Dari, Nepali, Russian, and Ukrainian.	https://nrcrim.org/toolkits/case-investigation-contact-tracing
Checklist: CICT among Refugees, Immigrants, and Migrants	This is a checklist of actions for health departments to consider when conducting CICT among refugee, immigrant, and migrant (RIM) communities.	https://z.umn.edu/6jfq
Checklist: Social Support Services for RIM Communities	This is a checklist of actions for health departments when organizing social service support among RIM communities - a key consideration for supporting communities with adherence to public health guidance, including CICT.	https://z.umn.edu/6jpn
Tips for Working with Interpreters during CICT	This document is for CICT professionals and outlines practical tips for working with interpreters.	https://z.umn.edu/6jpj
Build Your Own CICT Campaign	This guide supports individual leaders, community-based organizations, and local health departments to build effective COVID-19 messaging. Available in English and Spanish.	https://nrcrim.org/health-education/build-your-own-cict-campaign
“What is a Contact Tracer” Video Series	These videos describe the role of contact tracers and address common concerns among RIM communities. Available in Arabic, Burmese, Dari, Haitian Creole, Nepali, Pashto, Russian, Swahili, Tigrinya, and Ukrainian.	https://nrcrim.org/health-education/videos
Promising Practice: Outreach to RIM Communities ahead of CICT Efforts	This promising practice describes strategies to increase knowledge and trust in RIM communities before CICT begins.	https://nrcrim.org/outreach-rim-communities-ahead-cict-efforts
NRC-RIM Training for CICT Professionals	Training modules include “Working with RIM Communities in COVID-19,” “Working with Interpreters during CICT” and “Stress and Resilience for Case Investigators and Contact Tracers”	https://nrcrim.org/training/demand-training

NRC-RIM training modules were hosted on Canvas, an e-learning platform. Access to Canvas is free but requires users to register thus more information is available about users of the training modules. Over 1600 public health professionals representing all 50 states and DC have enrolled in NRC-RIM training modules. According to post module surveys, 73% of participants have demonstrated an increase in knowledge after completing a module and 94% of participants plan to apply the information they learned from the module in their day-to-day work.

## Discussion

At the beginning of the pandemic, NRC-RIM sought to enhance and expand on existing CICT programs by centering the perspectives of RIM communities and the case investigators and contact tracers that serve them. CICT will continue to be a core public health tool for COVID-19 response; communities with barriers to vaccination, including some RIM communities, will face greater disparities and will need CICT to prevent and mitigate outbreaks. A strong, culturally and linguistically affirming, CICT response is imperative to ensure that RIM communities receive the support and protection they need to continue to thrive.

These practices will have relevance beyond COVID-19. Community engagement, support for public health workers, and replicating the promising practices described here should be foundational elements of the public health response for any public health concern impacting RIM communities. All of the CICT resources developed for COVID-19 remain available on the NRC-RIM website to organizations to use. During the height of the Mpox outbreak in the United States, NRC-RIM worked to re-disseminate the CICT resources for use during Mpox response.

NRC-RIM has already leveraged the lessons learned from CICT activities to vaccination-related activities and resources that support engagement, partnerships and collaboration with RIM communities.
